# Switched and unswitched memory B cells detected during SARS-CoV-2 convalescence correlate with limited symptom duration

**DOI:** 10.1371/journal.pone.0244855

**Published:** 2021-01-28

**Authors:** Krista L. Newell, Deanna C. Clemmer, Justin B. Cox, Yetunde I. Kayode, Victoria Zoccoli-Rodriguez, Harry E. Taylor, Timothy P. Endy, Joel R. Wilmore, Gary M. Winslow

**Affiliations:** 1 Department of Microbiology and Immunology, Upstate Medical University, Syracuse, New York, United States of America; 2 Institute for Global Health and Translational Science, Upstate Medical University, Syracuse, New York, United States of America; Institut Cochin, FRANCE

## Abstract

Severe acute respiratory syndrome coronavirus-2 (SARS-CoV-2), the causative agent of the pandemic human respiratory illness COVID-19, is a global health emergency. While severe acute disease has been linked to an expansion of antibody-secreting plasmablasts, we sought to identify B cell responses that correlated with positive clinical outcomes in convalescent patients. We characterized the peripheral blood B cell immunophenotype and plasma antibody responses in 40 recovered non-hospitalized COVID-19 subjects that were enrolled as donors in a convalescent plasma treatment study. We observed a significant negative correlation between the frequency of peripheral blood memory B cells and the duration of symptoms for convalescent subjects. Memory B cell subsets in convalescent subjects were composed of classical CD24^+^ class-switched memory B cells, but also activated CD24-negative and natural unswitched CD27^+^ IgD^+^ IgM^+^ subsets. Memory B cell frequency was significantly correlated with both IgG1 and IgM responses to the SARS-CoV-2 spike protein receptor binding domain (RBD) in most seropositive subjects. IgM^+^ memory, but not switched memory, directly correlated with virus-specific antibody responses, and remained stable over 3 months. Our findings suggest that the frequency of memory B cells is a critical indicator of disease resolution, and that IgM^+^ memory B cells may play an important role in SARS-CoV-2 immunity.

## Introduction

There have now been over 36 million reported cases of SARS-CoV-2, including at least 1.05 million deaths worldwide [1]. As the work to develop effective vaccines and therapies to control the pandemic progresses, it is important to develop reliable approaches for assessing durable immunological memory. Identification of correlates of immunity to SARS-CoV-2 has been challenging, as clinical presentation and serological profiles vary between patients. Rare SARS-CoV-2-specific antibodies with potent neutralizing capacity have been isolated from recovered COVID-19 patients [2]. Additionally, acute COVID-19 patients have been observed to have perturbations of immune profiles, and have been grouped into three or more immunotype clusters [3]. On the basis of these findings, we focused on correlates of clinical outcomes in convalescent plasma donors that could be inferred through cell-based assays. Recent studies have correlated B cell responses in some individuals with immunity and protection [4].

B cells participate in the antiviral immune response by first rapidly releasing germline or near-germline antibodies from plasmablasts, via an extrafollicular pathway. Upon appropriate cytokine stimulation with or without T cell-mediated CD40 ligation, B cells undergo class switching and/or enter germinal centers within secondary lymphoid organs to undergo affinity maturation. This maturation process produces both long-lived plasma cells and memory B cells capable of responding to secondary challenge with homotypic or heterotypic antigenic challenge.

While many studies of the B cell response to SARS-CoV-2 have focused on plasmablast expansion, the benefit of this expansion has not been clear [3, 5, 6]. Indeed, clinical outcome correlations suggest that extrafollicular B cell activation and subsequent plasmablast generation are detrimental to host survival and COVID-19 symptom resolution. Memory B cells are also generated following SARS-CoV-2 infection [4]. B memory cells are found as multiple subsets, including canonical CD27^+^ class-switched memory B cells, but also activated CD24-negative and “innate-like” natural CD27^+^ IgD^+^ IgM^+^ subsets. Increased memory B cell frequencies can reveal a successful response to acute viral infection, and can provide information regarding the quality of cytokine production and T cell help during the acute immune response. In this study, we monitored memory B cell subsets and their relationship to clinical parameters in convalescent COVID-19 subjects. We provide evidence of stable B cell memory populations in recovered subjects that correlate with attenuated symptom duration. We propose that a well-developed memory B cell response provides a reliable measure of immunity that may also be useful for evaluating SARS-CoV-2 vaccine efficacy.

## Materials and methods

### Study design

Study participants were recruited at the SUNY Upstate Medical University Clinical Research Unit starting from March 2020 and is ongoing. Participants meeting eligibility criteria were adults aged 18 or older who have tested positive for SARS-CoV-2 and are at least 14 days past their first symptom. Exclusion criteria included the inability to give informed consent and/or an inability to donate plasma or blood transfusion in the past. Study participants were interviewed by study staff, and presented to the SUNY Upstate Clinical Research Unit for peripheral blood collection. Information regarding symptoms, including dates of first and last symptom, was self-reported. Donors were questioned about acute symptoms such as fever, shortness of breath, sore throat, cough that impacted activity, and fatigue that impacted activity. These indications were used to calculate dates of symptoms retrospectively. Lingering symptoms such as loss of taste and smell, mild cough or tickle in the throat, or lingering fatigue that did not impact their daily activity were not considered part of the acute illness and therefore not included in the length of illness. For donors reporting no symptoms, the date of positive RT-PCR test was used for the start and stop date of symptoms. These subjects were not included in correlative analysis of symptom duration. Seventeen of the original 40 convalescent plasma donors consented to a follow-up visit at 3 months. Two of these donors (one asymptomatic, one late convalescent) were excluded from analysis due to B cell abnormalities considered to be unrelated to SARS-CoV-2. Healthy control subjects were adults aged 18 or older who denied infection with or known exposure to SARS-CoV-2. Healthy controls were screened by anti-RBD plasma ELISA to confirm negative exposure status. Sample size was determined based on subject availability. All samples were de-identified following collection, and researchers conducting assays were blinded to clinical data until final comparative analysis.

### Blood sample processing and storage

PBMCs were obtained following gradient centrifugal separation of peripheral blood using Cell Preparation Tubes (CPT; BD Biosciences, Franklin Lakes, NJ, USA) for 30 minutes at 1700 x g. Plasma was separated, aliquoted, and stored at -20°C for antibody assays. The mononuclear layer was washed in PBS prior to counting on a Coulter particle counter (Beckman Coulter, Brea, CA, USA). PBMCs were either directly stained for flow cytometry (initial visit samples), or frozen slowly to -80°C in FBS and DMSO for short-term storage (3-month visit samples). Flow cytometry panel was validated using a sample of fresh vs. frozen PBMCs to ensure comparability in target detection.

### Flow cytometry

The following antibodies used for flow cytometry were obtained from BioLegend (San Diego, CA, USA): CD21 (Bu32), T-bet (4B10), CD38 (HIT2), CD11c (Bu15), CD3 (HIT3A), CD14 (HCD14), IgD (IA6-2), CD24 (MC5), IgM (MHM-88), CD27 (O323), and CD19 (HIB19). For flow cytometric analysis, single-cell suspensions were incubated with 1 microg/ml anti-CD16/CD32 in 2% normal goat serum/HBSS/0.1% sodium azide (in-house Fc blocking solution). The cells were then stained with aqua Live/Dead stain (Invitrogen, Carlsbad, CA, USA) and washed prior to incubation with mAbs. Cells were fixed, permeabilized, and stained for intracellular targets using an intracellular staining kit (BD Biosciences). Unstained controls were used to set the flow cytometer photomultiplier tube voltages, and single-color positive controls were used to adjust instrument compensation settings. Data from stained samples were acquired using a BD Fortessa flow cytometer equipped with DIVA software (BD Biosciences) and were analyzed using FlowJo™ Software (Becton, Dickinson and Company, Ashland, Oregon, USA). t-SNE visualization was generated using FlowJo™ automatic (opt-SNE) learning configuration, with 1000 iterations, a perplexity of 30, learning rate of 3179, exact KNN algorithm, and Barnes-Hut gradient algorithm.

### ELISA

Plasma samples were first heat-inactivated at 56°C for 30 mins before use in assays. Recombinant Twin-Strep-tagged RBD protein was purified from 293T cells transfected with the plasmid pαH-RBD SD1-3CH25, generously provided by Jason S. McLellan (University of Texas, USA), as previously described [7]. RBD protein was coated on Strep-Tactin® microplates plates (IBA Lifesciences, Göttingen, Germany) in binding buffer (100mM Tris pH 8, 150mM NaCl, 1mM EDTA) overnight at 4°C. Plates were washed three times with PBS-T (1x PBS/0.05% Tween-20) and subsequently blocked with 3% BSA (MilliporeSigma, St. Louis, MO, USA) in PBS-T for 1 hour at RT. Diluted plasma (1:40, 5-fold serially to 1:25 000) was loaded onto plates and incubated for 2 hours at RT. After incubation, plates were washed three times with PBS-T and HRP-conjugated secondary anti-human antibodies were used for detection. Total IgG was detected using goat-anti-human-IgG-HRP (Rockland Immunochemicals, Gilbertsville, PA, USA, #209–1304). IgM was detected using goat-anti-human-IgM-HRP (MilliporeSigma, #A6907), and IgG subclass antibodies were detected with mouse horseradish peroxidase (HRP)-conjugated anti-human IgG1 (9054–05), IgG2 (9060–05), IgG3 (9210–05), and IgG4 (9200–05) from SouthernBiotech (Birmingham, AL, USA). Wells were washed three times with PBS-T before the addition of HRP substrate SIGMAFAST OPD (MilliporeSigma, #P9187). Reaction was quenched with 1M hydrochloric acid (Fisher Scientific, Waltham, MA, USA, #S25856) and analyzed at 490 nM on a BioTek Synergy LX multi-mode plate reader. Area under the curve (AUC) analysis was performed using GraphPad Prism software (v8.4.3 for Mac, San Diego, California, USA), ignoring peaks with a height under 10% of the distance above baseline (Y = 0). Baseline was established by averaging the OD reading of all negative control plasma samples for the corresponding isotype. Negative controls were plasma from healthy control donors run in triplicate on each plate, and were validated against pre-COVID-19 pandemic plasma (prior to 2019). Both negative control and subject plasma samples were compared to matched serum to rule out interference due to plasma components. Plasma ELISAs for total human immunoglobulin isotype quantitation were performed using the Human Immunoglobulin Isotyping LEGENDplex 6-plex kit (BioLegend) according to the manufacturer’s instructions. Data were collected using a BD LSR II flow cytometer and analyzed using LEGENDplex Data Analysis Software.

### Statistical analyses

Statistical analyses were performed using GraphPad Prism software (v8.4.3). Analysis of correlation between flow cytometry, total serum immunoglobulin ELISA data, and continuous clinical data was performed using Pearson correlation coefficients for normally-distributed data sets, or nonparametric Spearman’s Rank correlation for data sets with fewer than 35 values. *p*-values are two-tailed and 95% confidence intervals shown where noted in figure legends. Statistical analysis of cell subset frequency between healthy and the total convalescent donor cohort from flow cytometry assays was performed using unpaired nonparametric Mann-Whitney test with two-tailed p-values and 95% confidence intervals. Multiple comparison analysis between each convalescent subject subgroup was done with Kruskal-Wallis test with Dunn’s correction. Adjusted p value was used to determine family-wise significance at alpha = 0.05. Statistical analysis of anti-RBD plasma ELISA data was performed using area under the curve (AUC) analysis within GraphPad Prism software (v8.4.3), ignoring peaks with a height under 10% of the distance above baseline (Y = 0). Baseline was established by averaging the OD reading of all negative control samples for the corresponding isotype. NS indicates a p value > 0.05, *p, < 0.05, **p, < 0.01, ***p, < 0.001, and ****p, <0.0001. The statistical tests performed are indicated in the figure legends. For column graphs, the column in each plot indicates the arithmetic mean of the dataset, and upper and lower bounds indicate SD of the dataset.

### Study approval

All participants provided written informed consent prior to participation in the study, which was performed according to a protocol approved by the Institutional Review Board (IRB) of the SUNY Upstate Medical University under IRB number 1587400. All clinical investigation was conducted according to Declaration of Helsinki principles.

### Graphics

S1A Fig was created using images from Servier Medical Art Commons Attribution 3.0 Unported License. (http://smart.servier.com).

## Results

### Longitudinal sampling of plasma donors with previous SARS-CoV-2 infection

To assess clinically-informative phenotypic characteristics of the B cell response in convalescent plasma donors, we evaluated peripheral blood PBMCs from this cohort and healthy volunteers by flow cytometry. Forty convalescent donors were recruited through the State University of New York Upstate Medical University Clinical Research Unit. They were sampled at an average of 69 days post-symptom onset, aged a mean of 51.6 years, and were composed of 35% males and 65% females. Donors and healthy controls were predominately white and non-Hispanic (S1 Fig). Convalescent donors were recruited following a positive SARS-CoV-2 PCR and at least 2 weeks following the last symptom, or for asymptomatic subjects, after a repeat SARS-CoV-2 PCR test that was negative. Fifteen subjects were also sampled at a 3-month follow-up visit, selected only for availability and consent. This latter subset included one asymptomatic subject, as well as those sampled in early, mid, and late convalescence. Healthy volunteers had a similar demographic profile to the convalescent cohort, and were considered healthy if reporting no known exposure to, or symptoms of, COVID-19. Characteristics of the study population and design are summarized in S2 Fig.

### B cell profiles varied widely during SARS-CoV-2 convalescence both between and within individuals

**Fig 1:**
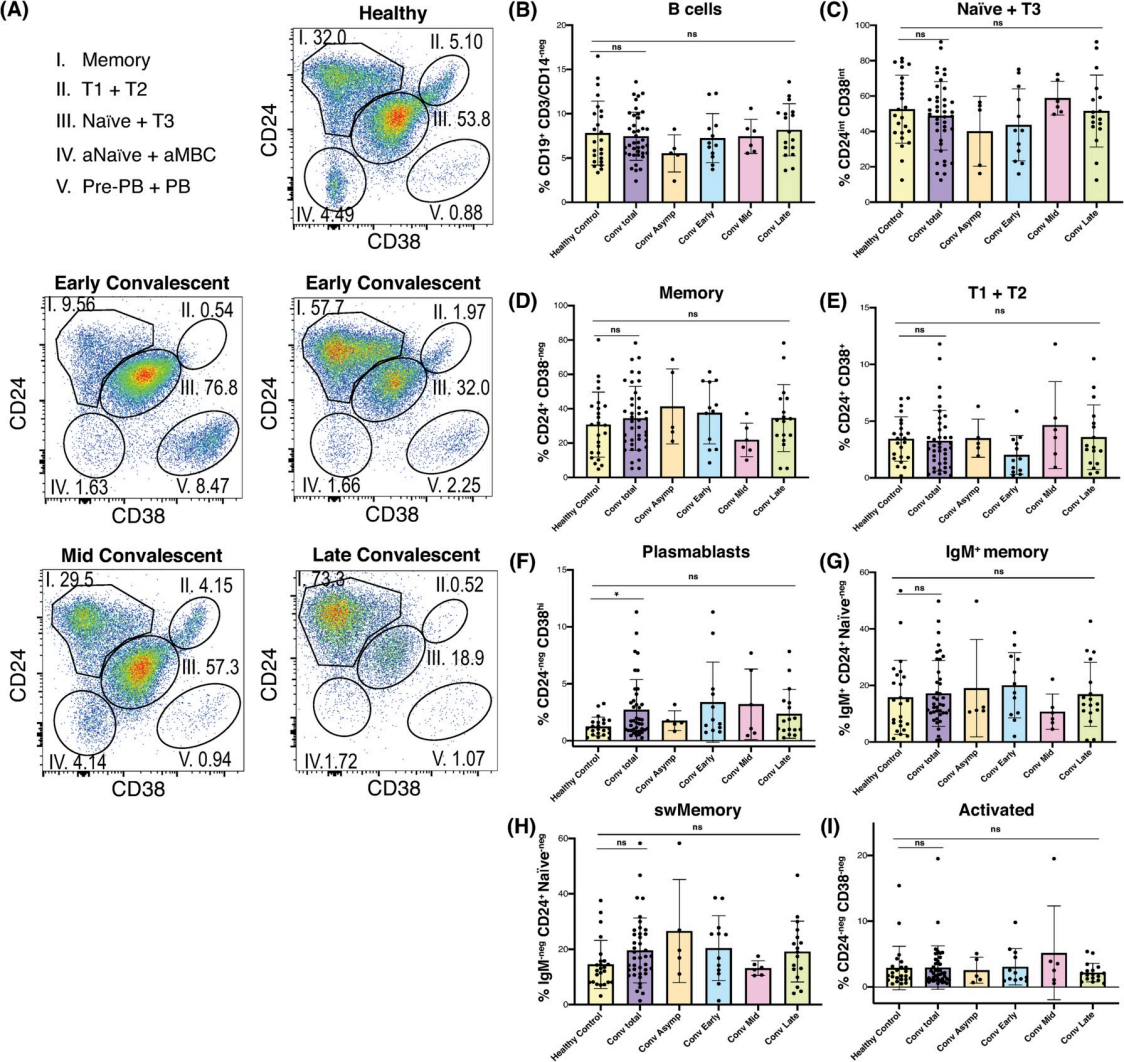
The B cell profiles of convalescent plasma donors were diverse. (A) Representative flow plots of live, singlet, CD19^+^ lymphocytes demonstrating 5 primary B cell subsets in one healthy and 4 convalescent subjects. (B-I) Histograms of healthy and convalescent donor frequencies of (B) total CD19^+^ B cells, and (C) naïve & transitional type 3, (D) memory, (E) transitional type 1 and 2, (F) plasmablast (G) IgM^+^ memory, (H) switched Memory, and (I) activated naïve/memory subsets among viable CD19^+^ lymphocytes. Bars represent mean ^+^/- SD. Statistical analysis between each donor subgroup was done with non-parametric Kruskal-Wallis test with Dunn’s correction for multiple comparisons. Adjusted p value was used to determine family-wise significance at alpha = 0.05. Healthy control and total convalescent groups also compared by Mann-Whitney test with two-tailed p value, alpha = 0.05. Healthy control *n* = 24, conv. total *n* = 40, asymp. *n* = 5, conv. early *n* = 12, conv. mid *n* = 6, conv. late *n* = 17, except for (E) and (F), where 1 and 2 healthy control statistical outliers were omitted, respectively.

As has been observed in other studies [2, 5], the B cell profiles of convalescent plasma donors were diverse. Using a well-defined gating strategy described by Sanz et al. [8] (S3 Fig), we identified convalescent subjects with expanded B cell memory, a robust plasmablast population, and a B cell phenotype resembling that of many healthy control samples (Fig 1A). We did not observe a difference in total CD19^+^ B cell frequency between convalescent and healthy subjects (Fig 1B), but as expected, convalescent naïve, transitional, and activated B cell frequencies followed an inverse trend relative to memory frequencies over time (Fig 1C–1E, 1G–1I). Memory B cell frequencies ranged widely, in both convalescent subjects and healthy controls (Fig 1D), potentially due to the minimal exclusion criteria used to select healthy volunteers. Despite this variation, convalescent subjects exhibited an inverse trend between naïve B cell and memory B cell frequencies, suggesting expansion of B cell memory in at least some subjects (Fig 1C and 1D). The plasmablast compartment was the only major B cell subset analyzed that remained significantly skewed in the convalescents, compared to healthy controls (Fig 1F). Moreover, elevated plasmablast frequencies were only observed in a subset of subjects. We observed that the majority of convalescent subjects had normal to elevated frequencies of both switched and unswitched CD38-negative CD24^+^ memory B cells (Fig 1G and 1H). These data contrast with observations of memory B cell loss in COVID-19 patients with acute severe disease [9], although we were unable to make conclusions regarding antigen-specific memory B cells in this study.

Given the demonstrated role of CD11c^+^ T-bet^+^ B cells in responses to viral antigens [10, 11], and their presence during acute SARS-CoV-2 infection [4], we screened convalescent donor samples for the presence of CD11c^+^ T-bet^+^ B cells. The frequency of both CD11c^+^ and T-bet^+^ B cells were only slightly elevated in convalescent subjects compared to healthy controls, likely reflecting a return to B cell homeostasis (S4A and S4B Fig). Likewise, significantly elevated frequencies of double-negative (DN) IgD-negative CD27-negative B cells were not observed in convalescent subjects (S4C Fig) in our cohort. These finding suggests that activated and DN populations may be preferentially involved in the early phase and/or critical clinical presentation of SARS-CoV-2 infection, as observed by Woodruff et al. [5] during acute severe COVID-19.

To further examine memory B cells in convalescent subjects, we visualized the flow cytometry datasets using an unbiased t-distributed stochastic neighbor embedding (t-SNE) algorithm (S4D Fig). This approach allowed us to resolve populations within clusters that may not be discrete using gating strategies alone, and to assess marker expression within these clusters. Using this approach, we observed that activated CD38-negative CD24-negative B cells were the primary clusters in which CD11c and T-bet were expressed (S4D Fig). These analyses reveal substantial heterogeneity not just in the B cell immunophenotype between subjects, but also within the B cell memory compartment itself.

### Shorter symptom duration was correlated with increased switched and IgM+ memory B cell frequencies in convalescent subjects

**Fig 2:**
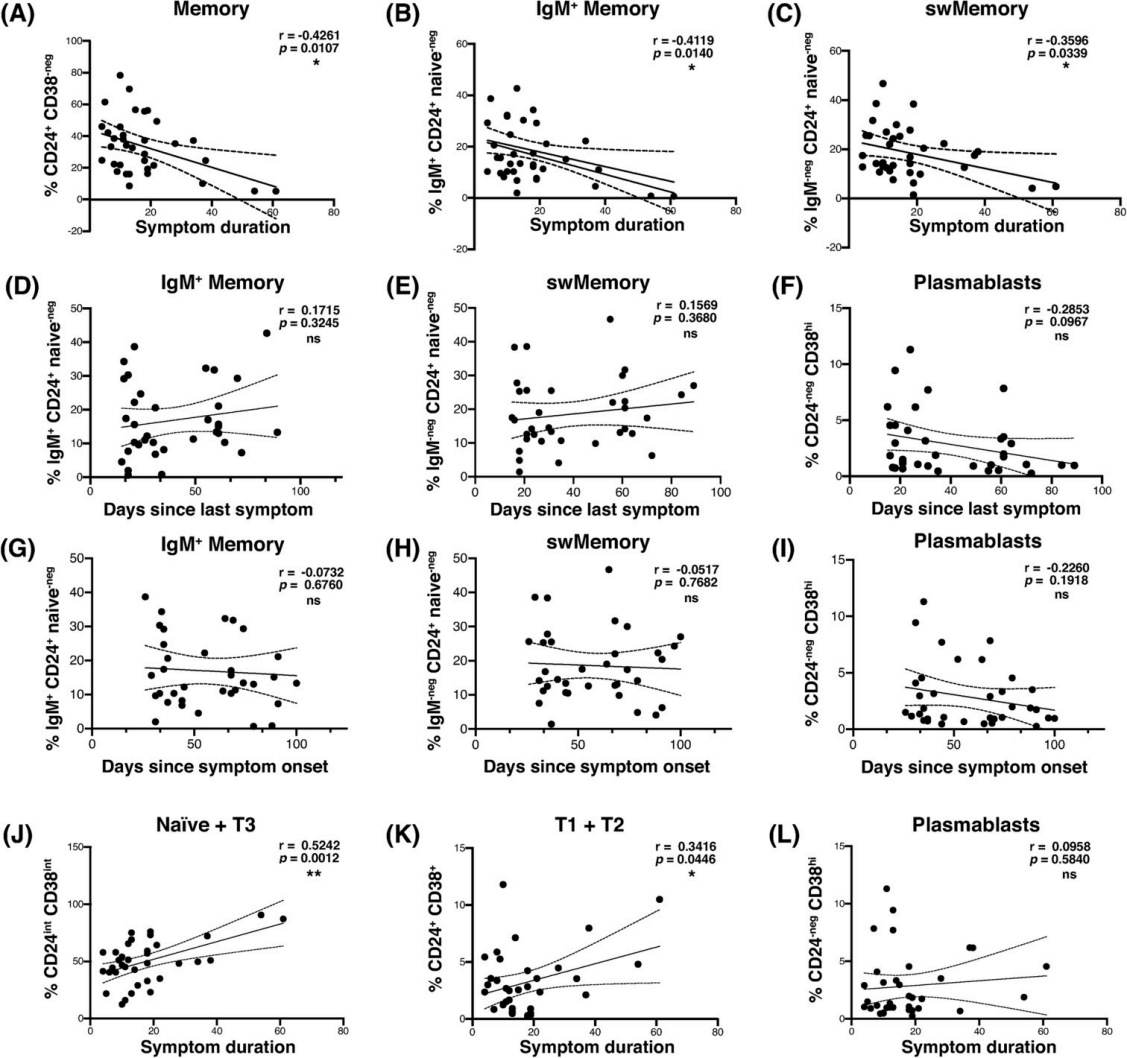
The frequency of memory B cells in the peripheral blood was correlated with shorter symptom duration following infection with SARS-CoV-2, and stable over time. (A-C) Scatterplot correlation of symptom duration in days, vs. the frequency among viable CD19^+^ B cells of (A) total memory, (B) IgM^+^ memory, and (C) switched memory. (D-F) Scatterplot correlation of days since last symptom vs. (D) IgM^+^ memory, (E) switched memory, and (F) plasmablast frequency among viable CD19^+^ lymphocytes. (G-I) Scatterplot correlation of days since symptom onset vs. (G) IgM^+^ memory, (H) switched memory, and (I) plasmablast frequency among viable CD19^+^ B cells. (J-L) Scatterplot correlation of symptom duration in days, vs. (J) naïve and transitional type 3, (K) transitional type 1 and 2, and (L) plasmablast frequency among viable CD19^+^ B cells. Pearson’s correlation r value and 95% confidence intervals shown with two-tailed p values, alpha = 0.05. *n* = 35 (all symptomatic subjects) for all.

While we observed diverse B cell subsets in convalescent subjects, it was still uncertain which, if any, of these subsets were correlated with clinical outcomes following symptomatic COVID-19 infection. An association between frequencies of memory B cells and enhanced recovery following COVID-19 pneumonia has been reported [9]. We therefore analyzed our B cell immunophenotyping dataset for its correlation with self-reported symptoms in our convalescent cohort. Our analysis revealed a significant negative correlation between the duration of COVID-19 symptoms and the frequency of memory B cells (Fig 2A). Similar correlations were observed for the IgM^+^ and switched memory B cell subsets (Fig 2B and 2C).

To determine whether these observations were due solely to the time of sampling, we analyzed the frequency of B cell subsets in convalescent subjects and the number of days between last symptom and sample collection, as well as the number of days between symptom onset and sampling. In these analyses, IgM^+^ and switched memory B cell frequencies were stable or enhanced over time, and we failed to observe any correlation between IgM^+^ or switched memory B cell frequency and the time since the last reported symptom (Fig 2D and 2E), or the time since symptom onset (Fig 2G and 2H). Longer symptom duration correlated with increased frequency of naïve and transitional B cells, though the reason for this relationship is unclear (Fig 2J and 2K). Neither age nor gender were observed to have a statistically significant influence on any of the clinical or immunophenotypic parameters examined in this dataset.

Several studies have reported robust expansion of peripheral blood plasmablasts during SARS-CoV-2 infection in some patients [5, 6]. In agreement with these data, our cohort of convalescent subjects contained individuals with dramatically elevated frequencies of CD38^hi^ CD24-negative CD19^+^ plasmablasts (see Fig 1F). Despite this trend, this subset was not significantly correlated with the duration of COVID-19 symptoms at the time point at which we sampled (Fig 2L). Plasmablast frequency among convalescent B cells did appear to wane over the time since last symptom, and symptom onset, confirming the contraction of the acute response (Fig 2F and 2I). Collectively, these data suggest that the presence of B cell memory, but not plasmablasts, is a clinical correlate of shorter duration of COVID-19 disease detectable during convalescence.

### Memory B cell frequency was correlated with anti-RBD antibody production in most seropositive convalescent subjects

**Fig 3:**
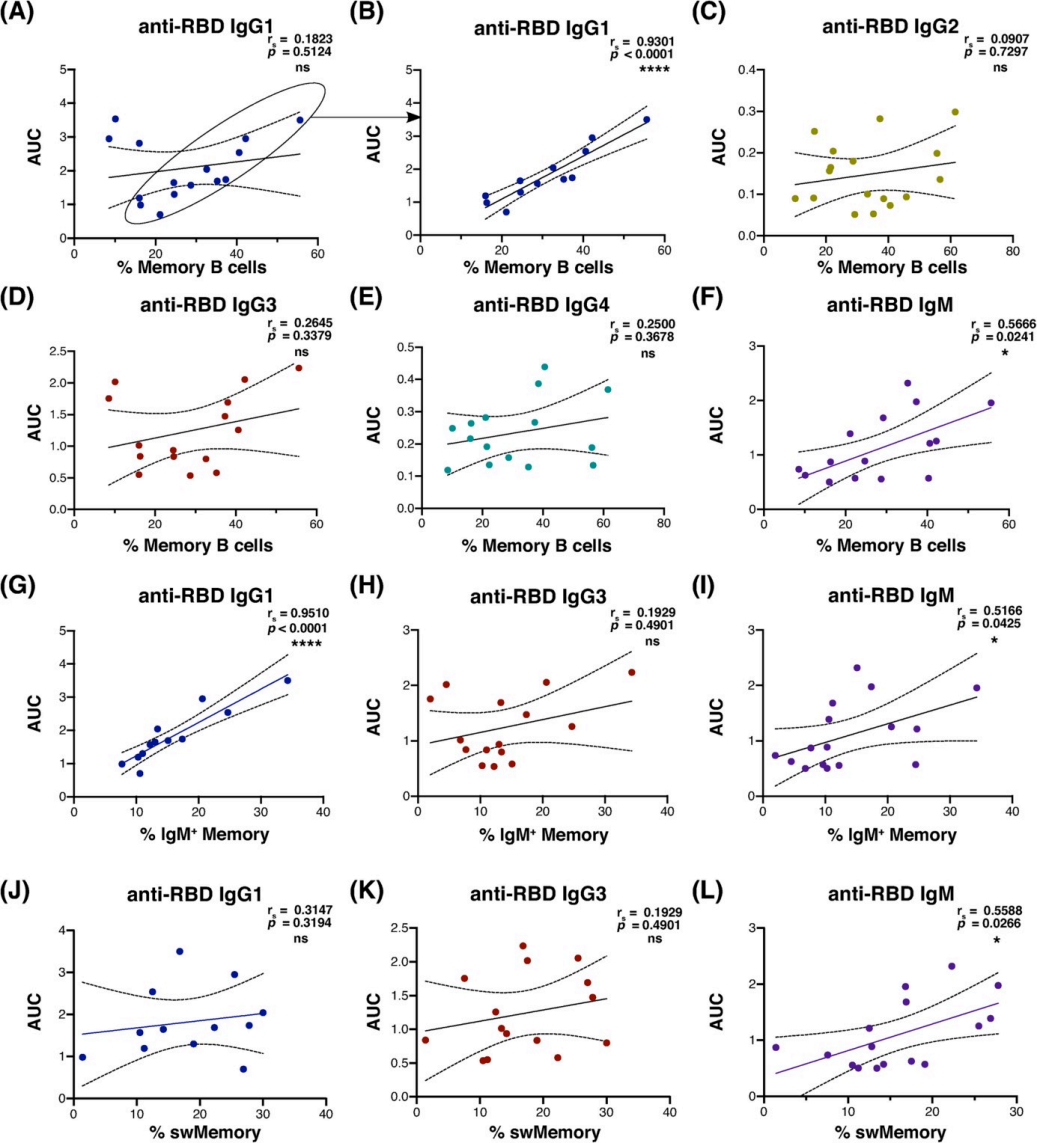
Anti-spike RBD antibody levels correlated with memory B cell frequency in most seropositive convalescent plasma donors. (A-F) Correlation of area under the curve for anti-RBD plasma absorbance vs. memory B cell frequency from convalescent subjects seropositive for each isotype (IgG1 & IgG3 *n* = 15, IgM *n* = 16). Subjects whose data points are circled in (A) were isolated from apparent outliers for statistical analysis of IgG1 in (B, G, and J; *n* = 12). (G-I) Scatterplot correlation of area under the curve for anti-RBD plasma absorbance vs. IgM^+^ memory B cell frequency among viable CD19^+^ lymphocytes. (J-L) Correlation of area under the curve for anti-RBD plasma absorbance vs. switched memory B cell frequency among viable CD19^+^ lymphocytes. Spearman’s correlation r_s_ value and 95% confidence intervals shown with two-tailed p value, alpha = 0.05 for all analyses. Regression lines shown to demonstrate trend only. Sample number distribution was the same between total, IgM^+^ and switched memory B cell analyses.

We next addressed whether the frequency of B cell memory in convalescent subjects correlated with the generation of anti-spike receptor-binding domain (RBD) antibodies. The spike RBD is thought to be required for SARS-CoV-2 binding and entry via the ACE2 receptor, and both inhibitory and neutralizing anti-RBD antibodies have been identified in infected and recovered subjects [4, 12]. In seropositive convalescent subjects, IgG1 anti-spike RBD was significantly correlated with CD24^+^ CD38-negative memory B cell frequency (Fig 3A, 3B and 3F) in most individuals, and with IgM anti-spike RBD for all seropositive subjects. This correlation was not observed for IgG2, IgG3, or IgG4 (Fig 3C–3E; for the full cohort, including subjects that were seronegative for each isotype, see S5 Fig). The 3 subjects with detectable IgG1 that did not correlate with memory B cell frequency all had relatively low proportions of memory B cells. Further studies of a larger sample group are warranted to explore potential causes of this grouping.

The positive relationship between IgG1 and total memory B cells held for IgM^+^ memory B cells (Fig 3G), but not for switched memory cells in most subjects (Fig 3J; for the full cohort including subjects that were seronegative for each isotype, see S6 Fig). For most anti-RBD IgG1-producing subjects, there was a nearly dose-dependent correlation between and anti-RBD IgG1 and IgM^+^ memory B cell frequency (Fig 3G). Anti-RBD IgM was significantly correlated with both switched and unswitched memory in these subjects, although not as markedly (Fig 3I and 3L). Memory B cell frequency and total immunoglobulin levels were not correlated, except for a weak positive correlation between memory and total IgG2 (S6G–S6L Fig). These results suggest that subjects with higher frequencies of IgM^+^ memory B cells may also generate a stronger SARS-CoV-2-reactive antibody response.

Among anti-RBD IgG1 seropositive subjects, we observed a clear negative trend between plasma anti-RBD IgG1 and symptom duration, although due to sample size limitations this effect was not statistically significant (S7A Fig). This trend was not observed for anti-RBD IgM in this group of subjects (S7B Fig). Within this IgG1-producing cohort, symptom duration also negatively correlated with both activated naïve/activated memory and T-bet+ B cell subsets, but positively correlated with age (S7D–S7F Fig). A correlation between circulating plasmablast frequency and symptom duration remained absent however (S7C Fig), likely due to the convalescent timepoint during which we sampled. Following the resolution of the acute response and clearance of virus, antibody production occurs largely via long-lived plasma cells, rather than short-lived circulating plasmablasts [4]. As our study only examined the peripheral blood, the majority of this bone marrow and secondary lymphoid organ-resident population would not have been identified. The association of T-bet+ and activated B cells with shorter symptom duration within anti-RBD IgG1 seropositive subjects aligns well with descriptions of this B cell subset having a low threshold for differentiation to the antibody-secreting cell fate [9]. Considered together, these results suggest that the apparent protective benefit of IgM^+^ memory B cells in limitation of symptom duration is mediated, at least in part by their contribution to, or parallel generation with, the SARS-CoV-2-reactive antibody response.

### Memory B cell frequencies were maintained or increased as plasmablasts returned to baseline

**Fig 4:**
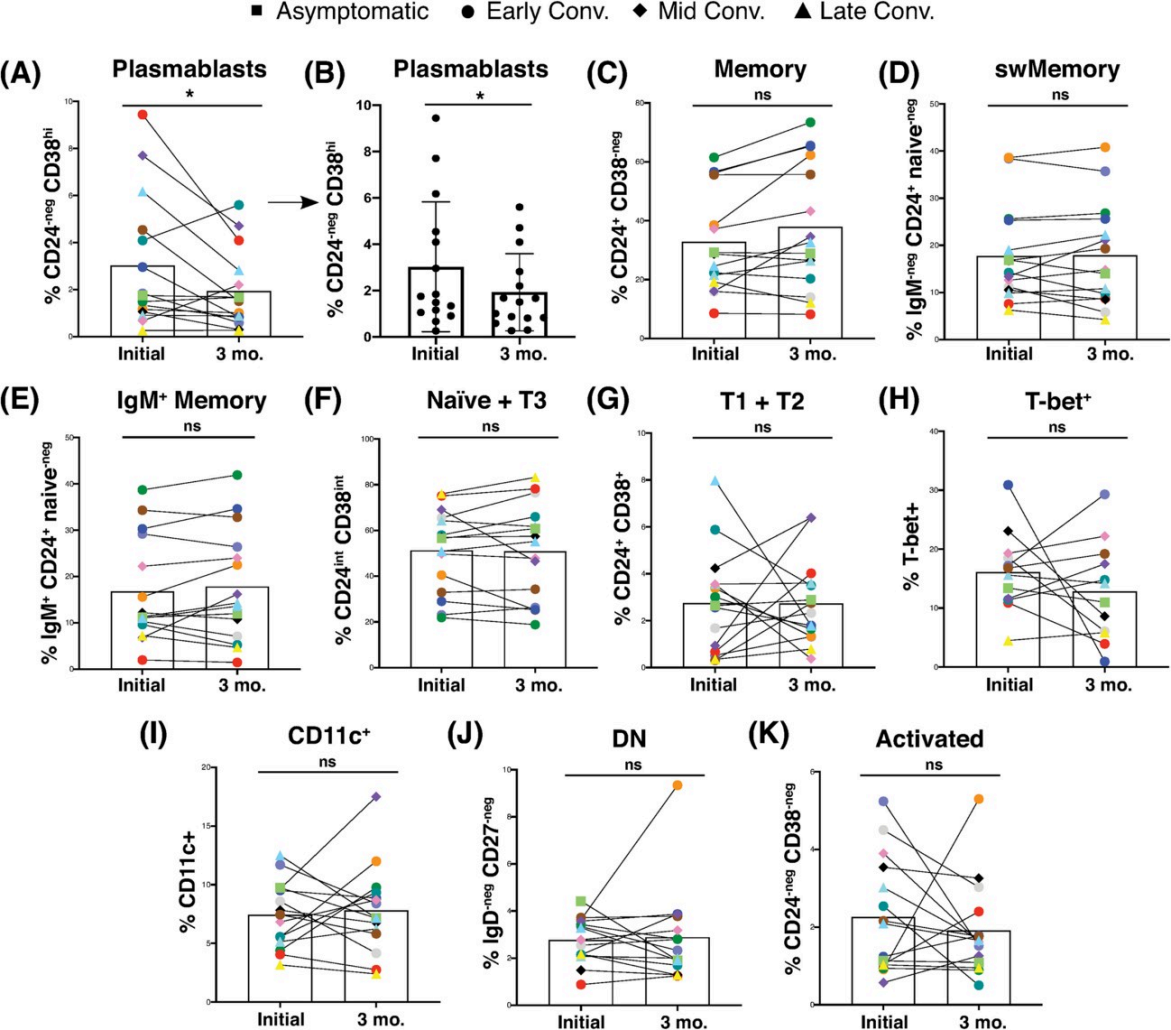
Convalescent SARS-CoV-2 subjects displayed a contraction of plasmablasts over time, but maintained memory B cells. (A-K) Frequency of (A and B) plasmablasts without and with SD, respectively, (C) total memory, (D) switched memory, (E) IgM^+^ memory, (F) naïve + transitional 3, (G) transitional 1 + 2, (H) T-bet^+^, (I) CD11c^+^, (J) DN, and (K) activated cells among CD19^+^ viable lymphocytes from 15 convalescent plasma donors at initial draw and 3-month follow-up visit. Follow-up donors were not selected, but the first available convalescent plasma donors to consent to a follow-up visit. Each pair of connected points (and color) represents an individual subject. Symbol shapes indicate convalescent subset. Bars represent mean, SD omitted for clarity in all except (B). Initial vs. 3-month intra-individual convalescent samples were compared by Wilcoxon matched-pairs signed rank test with two-tailed p value, alpha = 0.05.

We next addressed whether the memory B cells were a stable population, or waned, as did the plasmablast response. We re-sampled a subset of the convalescent cohort at least 3 months after the initial visit, based on availability and informed consent (S1 and S2 Figs). Results from this longitudinal analysis showed a significant contraction of the plasmablast response in the majority of subjects with a plasmablast frequency initially above 2%, as was expected during late convalescence (Fig 4A and 4B). We also observed that memory B cell frequencies and subset distribution were maintained or increased in most subjects (Fig 4C–4E). Naïve B cell frequency remained nearly constant (Fig 4F), and transitional subset frequencies fluctuated over time, without exhibiting a trend (Fig 4G). Although data from individual subjects demonstrated temporal variability with regards to the frequency of activation-associated B cell subsets, the frequency of T-bet^+^, CD11c^+^, DN, and activated B cells did not change significantly over time for the 3-month cohort as a whole (Fig 4H–4K). Additionally, these trends were not obviously impacted by the stage of convalescence when the first sample was obtained.

Of the B cell subsets analyzed, only plasmablasts exhibited a statistically significant change over the 3-month period wherein we assessed our convalescent cohort, suggesting a return to B cell homeostasis. This contraction is likely accompanied by the generation of germinal center-derived long-lived plasma cells, but that assessment is beyond the scope of this study of peripheral blood B cells. These findings suggest the potential for a role for switched and unswitched memory B cells in the maintenance of stable, durable SARS-CoV-2 immunity.

## Discussion

In this study we identified both unswitched and switched B cell memory as correlates of shorter COVID-19 symptom duration. Moreover, IgM^+^ memory correlated strongly with the anti-RBD IgG1 and IgM antibody response. These data suggest that a protective memory response occurs in at least some COVID-19 patients, preceded or accompanied by the generation of IgM^+^ memory B cells. We envision three possible explanations for these findings. First, it is possible that memory B cells identified in some individuals were generated in response to a previous coronavirus infection. Coronaviruses as a group likely generate cross-reactive B and T cells responses [13]. The observation that the anti-RBD IgG1 response was correlated with IgM^+^ memory cell frequency is paradoxical, given that IgM^+^ memory cells don’t produce switched immunoglobulin. We propose that IgM^+^ memory cells are generated in abundance during coronavirus infections, and that some of these undergo class switching with or without entering a germinal center reaction following a related coronavirus infection, thereby contributing to enhanced antigen-specific or cross-reactive IgG1 and IgM production. The capacity of IgM^+^ memory cells to preferentially enter germinal centers upon activation has been well-documented in mouse and human studies [14–16]. This characteristic versatility of IgM^+^ memory cells could be advantageous for immunity to pathogens, such as the coronaviruses, where infections with closely related strains often occur.

Alternatively, or coincidentally, the IgM^+^ memory B cells we identified in convalescent subjects could have developed in a T-independent manner from “MZ-like” B cells, with the promotion of IgG class-switch recombination driven by cytokines. Indeed, a large proportion of the IgM^+^ IgD^+^ memory B cells we identified in convalescent subjects expressed low levels of CD38, indicating that they may be re-circulating “MZ-like” B cells. Examination of this IgD^+^ IgM^+^ CD38^+/low^ CD24^+^ CD27^+^ B cell subset in isolation generated the same negative correlation with symptom duration, and positive correlation with anti-RBD IgG1 for seropositive subjects as observed for the total IgM^+^ memory B cell pool (data not shown). As natural or “innate-like” memory B cells are thought to be generated independently of germinal center reactions [17], our findings raise the important question as to whether natural, germinal center- and possibly T cell-independent B cell memory contributes to protection during SARS-CoV-2.

The lack of correlation between the frequency of resting memory B cells and CD11c^+^ and/or T-bet^+^ B cells in the full cohort of convalescent subjects was unexpected, given the pivotal role these molecules play in type-1 B cell immunity [10, 11, 18]. We hypothesize that these cells play a key role during the acute phase and during chronic viral infections, but are not essential during the convalescent phase of SARS-CoV-2 infection where antigen is unlikely to be present. The negative correlation between symptom duration, and T-bet+ and activated B cells observed in anti-RBD IgG1-producing subjects suggests that these cells may have contributed to protection during the acute response, but also intimates that antigen persistence may vary between individuals during convalescence. Additional prospective studies and kinetic analyses of previously-exposed and naïve individuals will help to resolve this question.

A second explanation for the enhancement of COVID-19 recovery coincident with memory B cell expansion, is that naïve subjects whose B cells received more efficient T cell help during primary infection generated a larger pool of memory B cells. This explanation is consistent with the close relationship between memory and pathogen-specific antibody production we observed. This explanation would suggest that T cells contributed to a better germinal center response in some individuals. In contrast, subjects whose B cells received insufficient or inappropriate T cell help would generate poor germinal center reactions, fewer antigen-specific antibodies, and have a longer symptomatic disease period.

T cell help occurs largely within germinal centers, suggesting that the local immune environment may influence the B cell response to SARS-CoV-2. Indeed, severe disruption of lymphatic tissue organization and germinal center formation have been observed in severe COVID-19 cases [19]. Moreover, studies from our laboratory have shown that follicle architecture disruption, acute plasmablast expansion, and type-1 cytokine skewing occurs during murine intracellular bacterial infection [20–22]. We observed IgM^+^ memory to be protective during *E*. *muris* infection, despite severe immunopathology associated with primary infection. In this way, it is possible that during mild infection, low levels of the inflammatory cytokines thought to cause germinal center disruption during severe SARS-CoV-2 infection contribute to the generation of protective IgM^+^ B cell memory in a GC-dependent or GC-independent manner. The data herein suggests that IgM^+^ memory B cells may complement long-lived plasma cells and provide protection against subsequent SARS-CoV infection.

Finally, the benefit of memory B cell expansion may only be an indication that acute inflammation and excessive cytokine production did not occur in some individuals. Under inflammatory conditions, such as those occurring in systemic lupus erythematous (SLE), B cell subsets have been shown to be skewed toward an activated, extrafollicular effector fate [23]. It has been suggested that B cell responses to severe acute COVID-19 disease have similar characteristics to those observed in SLE [5]. The variability in the penetrance of this phenotype may be explained by the influence of gender and pre-existing autoimmunity. Future work using animal models of COVID-19 may help to resolve these questions. Additionally, we were unable to recruit a racially-diverse study cohort for both convalescent and healthy subjects. Considering the known disparities in SARS-CoV-2 disease severity between racial backgrounds, this limitation should be taken into consideration in application of our findings.

The retrospective and descriptive nature of our study should also be considered, including the variation in time between last symptom and sample draw. While we attempted to address this variable by examining B cell subset frequencies over time, it was not possible to completely isolate the contribution of time to our findings with the available sample size and study design. Indeed, changes in the B cell response over time may have contributed to the correlations between antibody responses and memory B cell frequencies in some individuals. Nevertheless, our data support the consideration of B cell memory as a measure of SARS-CoV-2 protection, even if the effect is indirect.

## Conclusions

Our studies support other work that has reported that stable populations of memory B cells are likely generated following SARS-CoV-2 infection, and that these memory B cells are a correlate of an effective primary response [4, 9]. These studies challenge early claims of waning immunity shortly after SARS-CoV-2 infection. Our data also show that some individuals may have better natural immunity, as the frequency of memory B cells was widely variable among both healthy controls and convalescent donors. While we cannot exclude the possibility of unreported non-SARS-CoV-2 infection or pathogenic immune activation in our healthy donor cohort, we consider this unlikely to contribute significantly. We also propose that both IgM^+^ and switched memory B cells may provide a good indication of vaccine efficacy, and that individuals with large numbers of IgM^+^ memory B cells may be better protected from future re-infection with homotypic or heterotypic infection. Our findings suggest that IgM^+^ memory B cells are central to the COVID-19 adaptive immune response, and highlight the need for prospective SARS-CoV-2 studies to determine whether large memory B cell populations are a pre-existing correlate of protection, or a durable measure of the antiviral immune response.

## Supporting information

S1 Fig Demographic data. (A-C) Distribution of (A) gender, (B) age, and (C) ethnicity among the convalescent plasma donor cohort (*n* = 40). (D-F) Distribution of (D) gender, (E) age, and (F) ethnicity among healthy donors (*n* = 24). (G-H) Distribution of (G) gender and (H) age among the subset convalescent plasma donor cohort (*n* = 15) analyzed in Fig 4, three months after initial visit. (I) Cumulative table of demographic data.
(PDF)

S2 Fig Study design and clinical data. (A) Graphical representation of convalescent subject groupings and gender (*n* = 40). All grouping and subset designations were done retrospectively. (B) Symptom and sampling timeline for symptomatic subjects (*n* = 35), ordered by length of convalescence.
(PDF)

S3 Fig Flow cytometry gating strategies for B cell subset analysis. (A) Representative plots demonstrating gating strategy for flow cytometric identification of B cell subsets. (B) Flow chart showing the dichotomy and subset discrimination used to assess B cell subsets from peripheral blood PBMCs.
(PDF)

S4 Fig B cell subset and cluster analysis. (A-C) Healthy and convalescent donor cell frequencies for (A) CD11c+, (B) T-bet+, and (C) DN subsets among viable CD19+ lymphocytes. Bars represent mean +/- SD. Statistical analysis between each donor subgroup was done with non-parametric Kruskal-Wallis test with Dunn’s correction for multiple comparisons. Adjusted p value was used to determine family-wise significance at alpha = 0.05. Healthy control and total convalescent groups also compared by Mann-Whitney test with two-tailed p value, alpha = 0.05. Healthy control *n* = 24, conv. total *n* = 40, asymp. *n* = 5, conv. early *n* = 12, conv. mid *n* = 6, conv. late *n* = 17, except for T-bet (B), where *n* = 4, 9, 6, and 16, respectively, due to technical limitations. (D) tSNE representation of flow cytometry data sub-gated to highlight key clusters of viable CD19+ lymphocytes from a representative convalescent donor with an enriched memory B cell subset. Heatmap overlay shows median expression for the target below each plot.
(PDF)

S5 Fig Symptom duration vs. anti-spike RBD-specific and total antibody levels in full cohort of convalescent plasma donors. (A-E) Scatterplot correlation of area under the curve for plasma anti-RBD absorbance vs. symptom duration for individual Ig isotypes and subclasses. (F-K) Scatterplot correlation of total plasma antibody concentration vs. symptom duration for individual Ig isotypes and subclasses. Pearson’s correlation *r* value and 95% confidence intervals shown with two-tailed p value, alpha = 0.05. *n* = 35 (all symptomatic subjects) for all plots.
(PDF)

S6 Fig Memory B cell frequency vs. anti-RBD antibody and total immunoglobulin in full cohort of convalescent plasma donors. (A-E) Scatterplot correlation of area under the curve for anti-RBD plasma antibody absorbance vs. memory B cell frequency for individual Ig isotypes and subclasses. (G-L) Scatterplot correlation of total plasma antibody concentration vs. memory B cell frequency from all convalescent subjects. Units reflect relative abundance of isotype, and were adjusted for graphical consistency. Pearson’s correlation *r* value and 95% confidence intervals shown with two-tailed p value, alpha = 0.05. *n* = 40 for all anti-RBD plots, *n* = 39 for total Ig plots (one asymptomatic subject was not tested).
(PDF)

S7 Fig Symptom duration correlations in anti-RBD IgG1-producing convalescent plasma donors. (A-B) Scatterplot correlation of area under the curve for plasma anti-RBD (A) IgG1 and (B) IgM absorbance vs. symptom duration. (C) Scatterplot correlation of symptom duration in days vs. frequency of (C) plasmablasts, (D) activated naive/memory, and (E) T-bet+ cells among CD19+ B cells. Scatterplot correlation of symptom duration vs. age in years. n = 11 for all plots, circled in Fig 3A. Spearman’s correlation r_s_ value and 95% confidence intervals shown with two-tailed p value, alpha = 0.05 for all analyses. Regression lines shown to demonstrate trend only.
(PDF)
