# Supplementary material for: Switched and unswitched memory B cells detected during SARS-CoV-2 convalescence correlate with limited symptom duration
Source: PLoS One. 2021 Jan 28;16(1):e0244855. doi: 10.1371/journal.pone.0244855 (PMC7843013; doi:10.1371/journal.pone.0244855)
Supplement: S1 Fig — (A-C) Distribution of (A) gender, (B) age, and (C) ethnicity among the convalescent plasma donor cohort (n = 40). (D-F) Distribution of (D) gender, (E) age, and (F) ethnicity among healthy donors (n = 24). (G-H) Distribution of (G) gender and (H) age among the subset convalescent plasma donor cohort (n = 15) analyzed in Fig 4, three months after initial visit. (I) Cumulative table of demographic data. (PDF) [file pone.0244855.s001.pdf]

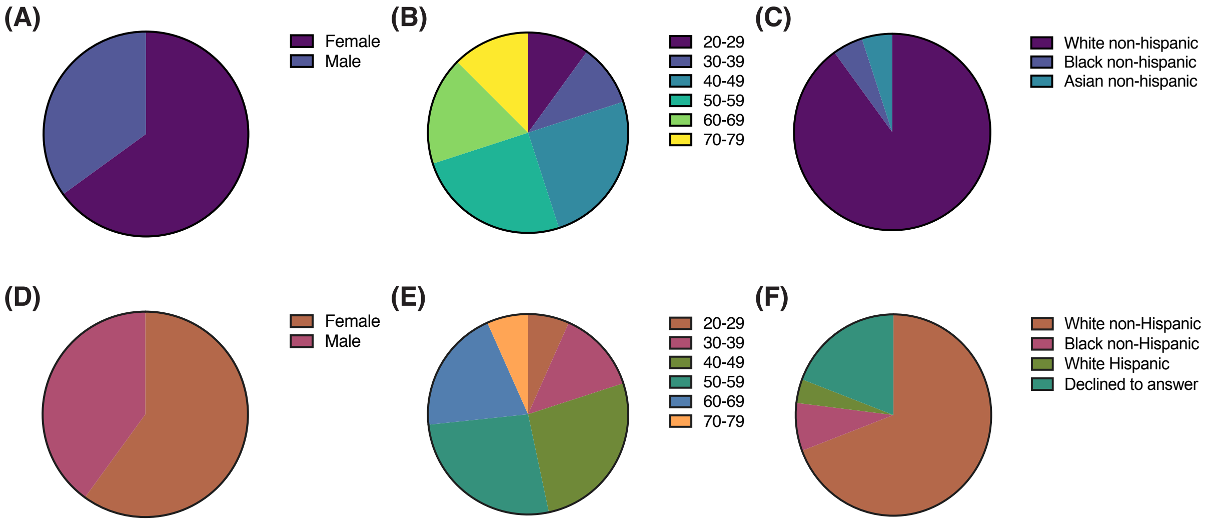

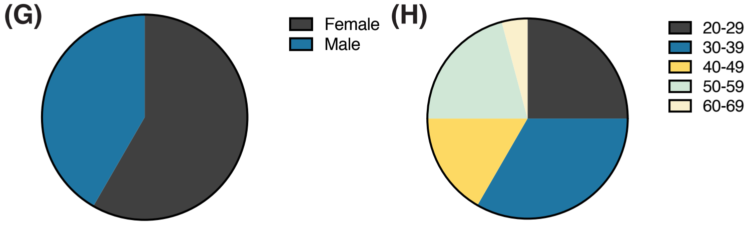

(I)

	#/% Convalescent subjects (n = 40)	#/% of 3 mo. subjects (n = 15)	#/% Healthy subjects (n = 24)
**Gender**			
Female	26/65	9/60.0	14/58.3
Male	14/35	6/40.0	10/41.7
**Age group**			
20-29	4/10	1/6.7	6/25
30-39	4/10	2/13.3	8/33.3
40-49	10/25	4/26.7	4/16.7
50-59	10/25	4/26.7	5/20.8
60-69	7/17.5	3/20.0	1/4.2
70-79	5/12.5	1/6.7	0/0
**Ethnicity**			
White non-Hispanic	36/90	15/100	18/75
Black non-Hispanic	2/5	0/0	2/8.3
Asian non-Hispanic	2/5	0/0	0/0
White Hispanic	0/0	0/0	1/4.2
Declined to answer	0/0	0/0	5/20.8

S1 Fig. Demographic data. (A-C) Distribution of (A) gender, (B) age, and (C) ethnicity among the convalescent plasma donor cohort ($n = 40$). (D-F) Distribution of (D) gender, (E) age, and (F) ethnicity among healthy donors ($n = 24$). (G-H) Distribution of (G) gender and (H) age among the subset convalescent plasma donor cohort ($n = 15$) analyzed in fig 4, three months after initial visit. (I) Cumulative table of demographic data.
