# Supplementary material for: Switched and unswitched memory B cells detected during SARS-CoV-2 convalescence correlate with limited symptom duration
Source: PLoS One. 2021 Jan 28;16(1):e0244855. doi: 10.1371/journal.pone.0244855 (PMC7843013; doi:10.1371/journal.pone.0244855)
Supplement: S2 Fig — (A) Graphical representation of convalescent subject groupings and gender (n = 40). All grouping and subset designations were done retrospectively. (B) Symptom and sampling timeline for symptomatic subjects (n = 35), ordered by length of convalescence. (PDF) [file pone.0244855.s002.pdf]

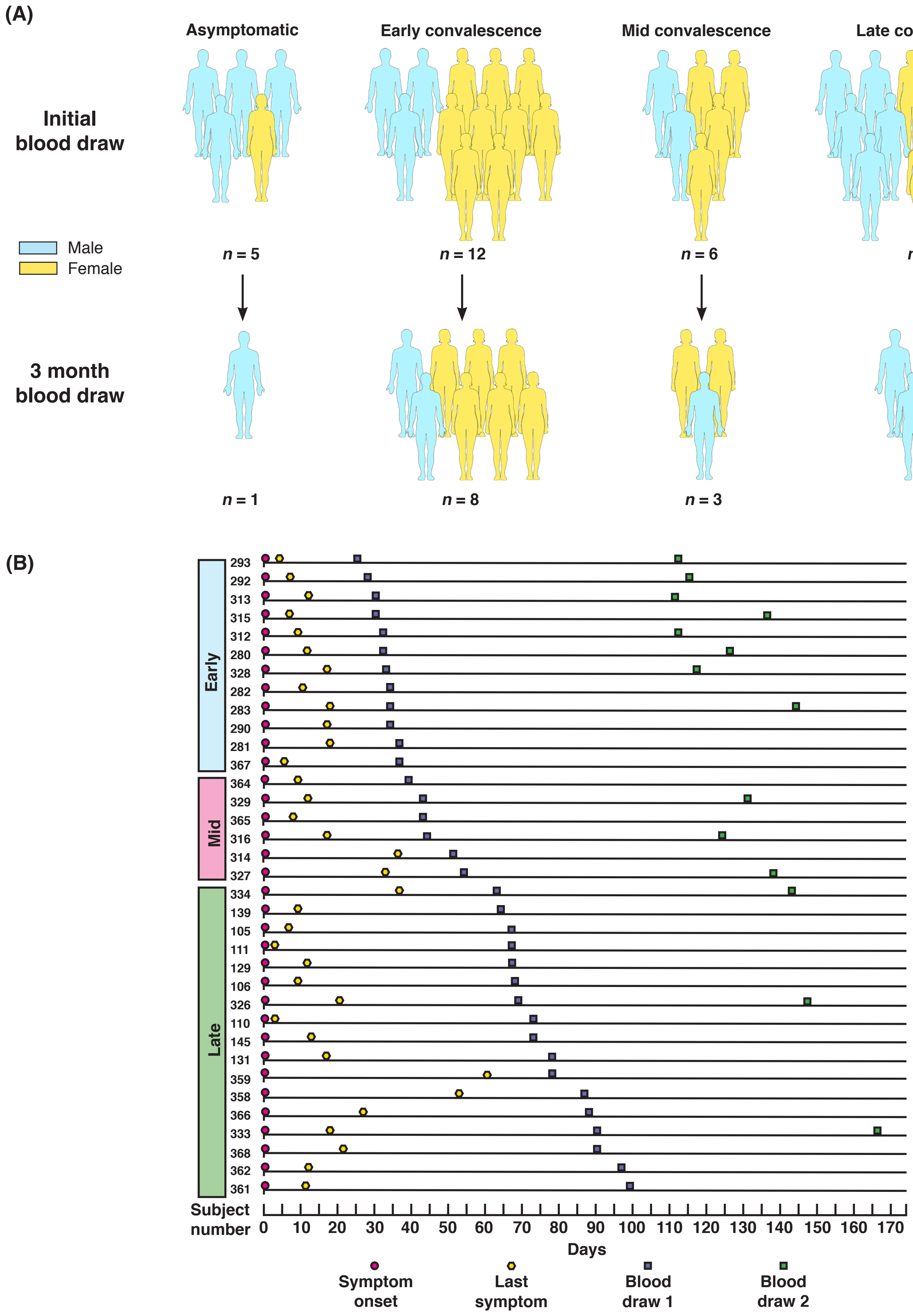

S2 Fig. Study design and clinical data. (A) Graphical representation of convalescent subject groupings and gender ($n = 40$). All grouping and subset designations were done retrospectively. (B) Symptom and sampling timeline for symptomatic subjects ($n = 35$), ordered by length of convalescence.
