# Supplementary material for: Switched and unswitched memory B cells detected during SARS-CoV-2 convalescence correlate with limited symptom duration
Source: PLoS One. 2021 Jan 28;16(1):e0244855. doi: 10.1371/journal.pone.0244855 (PMC7843013; doi:10.1371/journal.pone.0244855)
Supplement: S3 Fig — (A) Representative plots demonstrating gating strategy for flow cytometric identification of B cell subsets. (B) Flow chart showing the dichotomy and subset discrimination used to assess B cell subsets from peripheral blood PBMCs. (PDF) [file pone.0244855.s003.pdf]

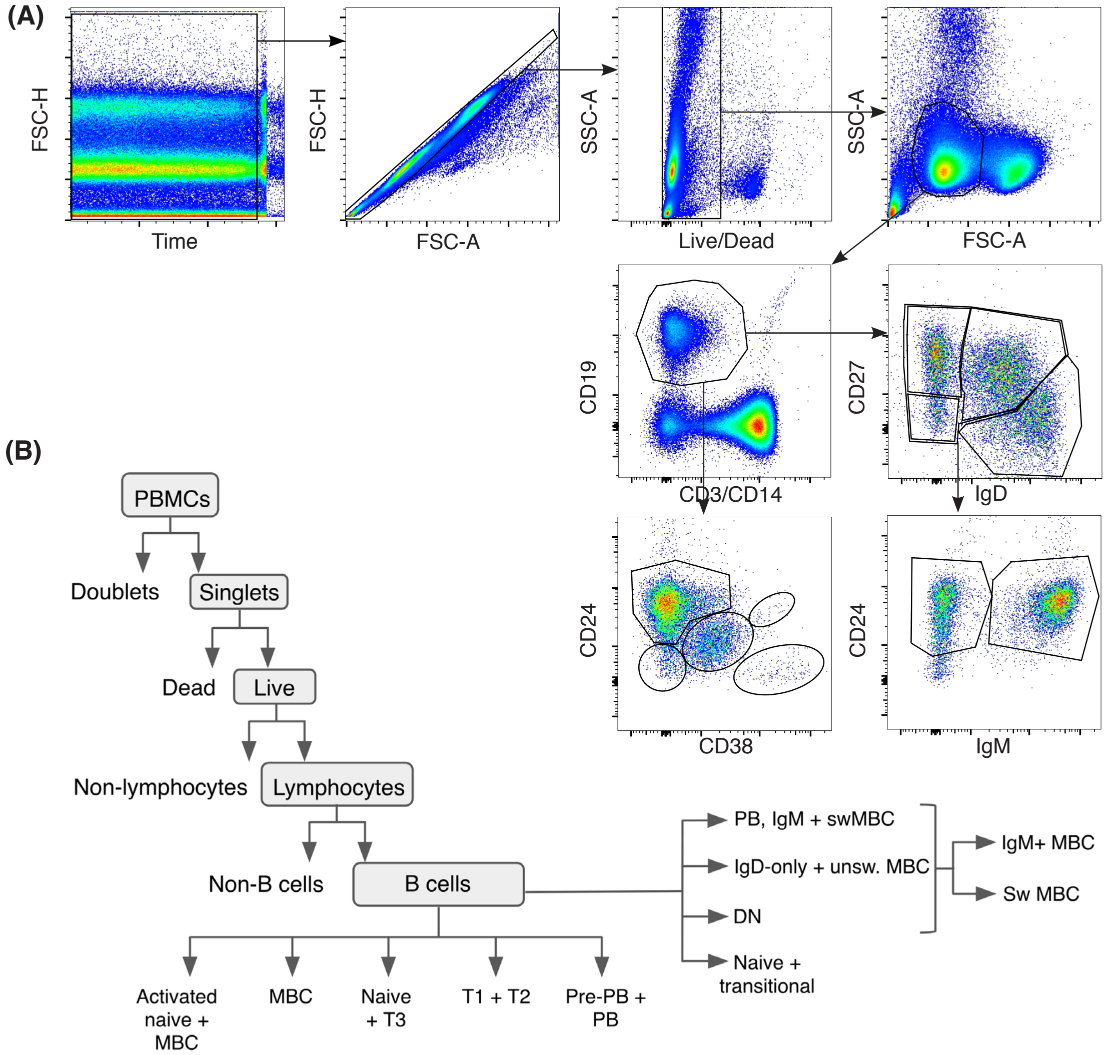

S3 Fig. Flow cytometry gating strategies for B cell subset analysis. (A) Representative plots demonstrating gating strategy for flow cytometric identification of B cell subsets. (B) Flow chart showing the dichotomy and subset discrimination used to assess B cell subsets from peripheral blood PBMCs.
