# Supplementary material for: Switched and unswitched memory B cells detected during SARS-CoV-2 convalescence correlate with limited symptom duration
Source: PLoS One. 2021 Jan 28;16(1):e0244855. doi: 10.1371/journal.pone.0244855 (PMC7843013; doi:10.1371/journal.pone.0244855)
Supplement: S4 Fig — (A-C) Healthy and convalescent donor cell frequencies for (A) CD11c+, (B) T-bet+, and (C) DN subsets among viable CD19+ lymphocytes. Bars represent mean +/- SD. Statistical analysis between each donor subgroup was done with non-parametric Kruskal-Wallis test with Dunn’s correction for multiple comparisons. Adjusted p value was used to determine family-wise significance at alpha = 0.05. Healthy control and total convalescent groups also compared by Mann-Whitney test with two-tailed p value, alpha = 0.05. Healthy control n = 24, conv. total n = 40, asymp. n = 5, conv. early n = 12, conv. mid n = 6, conv. late n = 17, except for T-bet (B), where n = 4, 9, 6, and 16, respectively, due to technical limitations. (D) tSNE representation of flow cytometry data sub-gated to highlight key clusters of viable CD19+ lymphocytes from a representative convalescent donor with an enriched memory B cell subset. Heatmap overlay shows median expression for the target below each plot. (PDF) [file pone.0244855.s004.pdf]

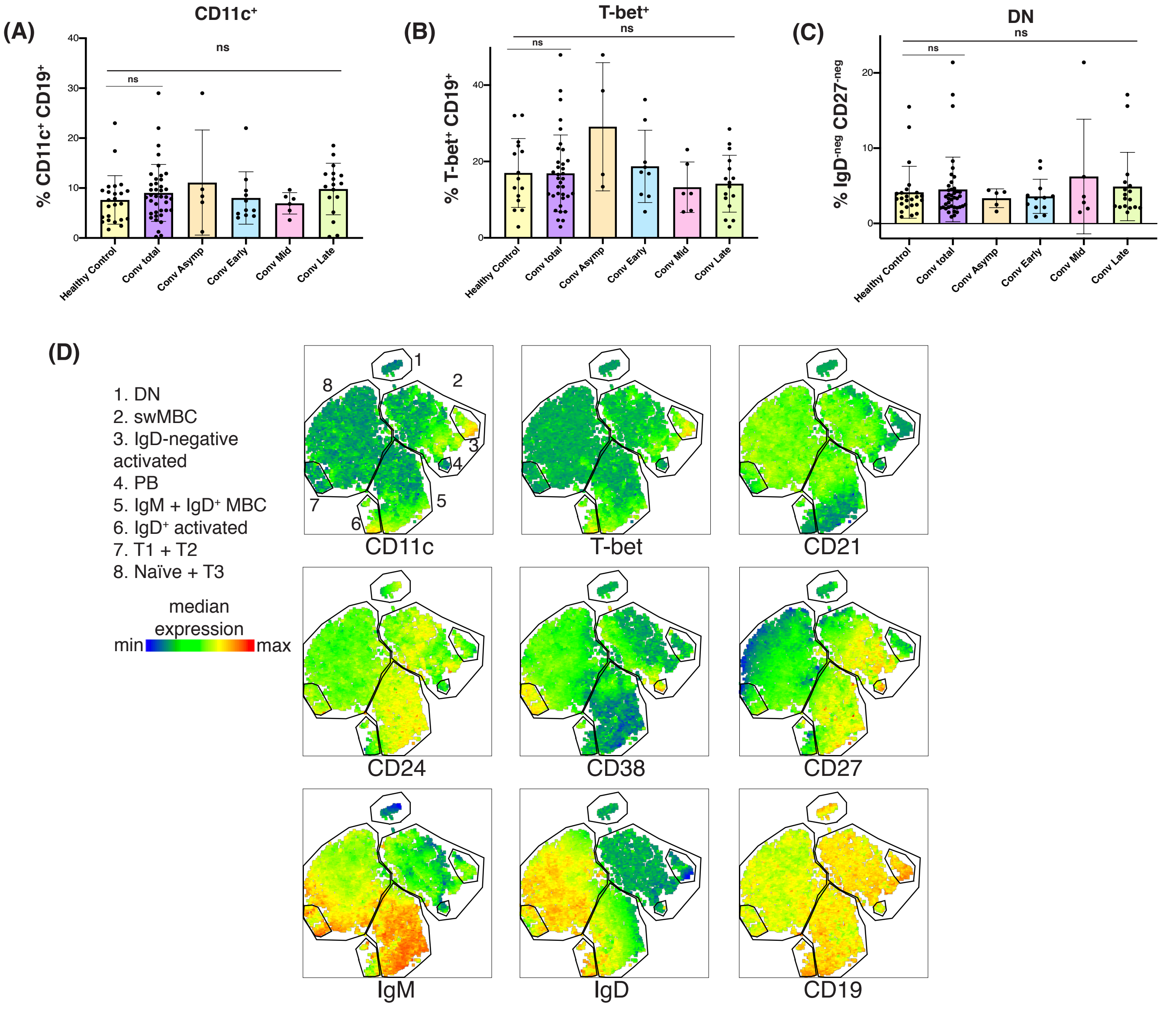

S4 Fig. B cell subset and cluster analysis. (A-C) Healthy and convalescent donor cell frequencies for (A) CD11c+, (B) T-bet+, and (C) DN subsets among viable CD19+ lymphocytes. Bars represent mean +/- SD. Statistical analysis between each donor sbgroup was done with non-parametric Kruskal-Wallis test with Dunn's correction for multiple comparisons. Adjusted *p* value was used to determine family-wise significance at alpha = 0.05. Healthy control and total convalescent groups also compared by Mann-Whitney test with two-tailed *p* value, alpha = 0.05. Healthy control $n = 24$, conv. total $n = 40$, asymp. $n = 5$, conv. early $n = 12$, conv. mid $n = 6$, conv. late $n = 17$, except for T-bet (B), where $n = 4$, 9, 6, and 16, respectively, due to technical limitations. (D) tSNE representation of flow cytometry data sub-gated to highlight key clusters of viable CD19⁺ lymphocytes from a representative convalescent donor with an enriched memory B cell subset. Heatmap overlay shows median expression for the target below each plot.
