# Supplementary material for: Switched and unswitched memory B cells detected during SARS-CoV-2 convalescence correlate with limited symptom duration
Source: PLoS One. 2021 Jan 28;16(1):e0244855. doi: 10.1371/journal.pone.0244855 (PMC7843013; doi:10.1371/journal.pone.0244855)
Supplement: S5 Fig — (A-E) Scatterplot correlation of area under the curve for plasma anti-RBD absorbance vs. symptom duration for individual Ig isotypes and subclasses. (F-K) Scatterplot correlation of total plasma antibody concentration vs. symptom duration for individual Ig isotypes and subclasses. Pearson’s correlation r value and 95% confidence intervals shown with two-tailed p value, alpha = 0.05. n = 35 (all symptomatic subjects) for all plots. (PDF) [file pone.0244855.s005.pdf]

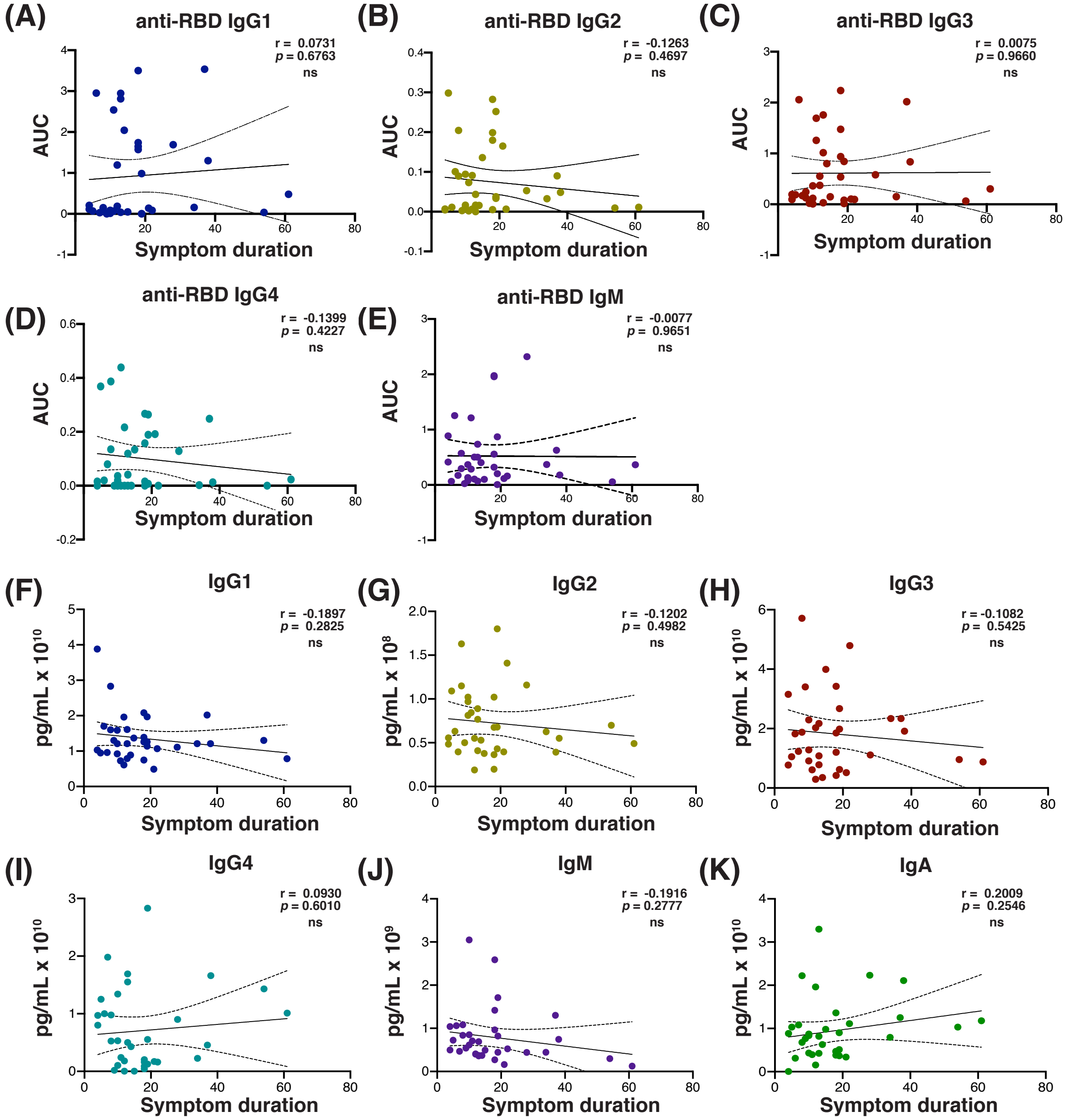

S5 Fig. Symptom duration vs. anti-spike RBD-specific and total antibody levels in full cohort of convalescent plasma donors. (A-E) Scatterplot correlation of area under the curve for plasma anti-RBD absorbance vs. symptom duration for individual Ig isotypes and subclasses. (F-K) Scatterplot correlation of total plasma antibody concentration vs. symptom duration for individual Ig isotypes and subclasses. Pearson's correlation r value and 95% confidence intervals shown with two-tailed *p* value, alpha = 0.05. *n* = 35 (all symptomatic subjects) for all plots.
