# Supplementary material for: Switched and unswitched memory B cells detected during SARS-CoV-2 convalescence correlate with limited symptom duration
Source: PLoS One. 2021 Jan 28;16(1):e0244855. doi: 10.1371/journal.pone.0244855 (PMC7843013; doi:10.1371/journal.pone.0244855)
Supplement: S6 Fig — (A-E) Scatterplot correlation of area under the curve for anti-RBD plasma antibody absorbance vs. memory B cell frequency for individual Ig isotypes and subclasses. (G-L) Scatterplot correlation of total plasma antibody concentration vs. memory B cell frequency from all convalescent subjects. Units reflect relative abundance of isotype, and were adjusted for graphical consistency. Pearson’s correlation r value and 95% confidence intervals shown with two-tailed p value, alpha = 0.05. n = 40 for all anti-RBD plots, n = 39 for total Ig plots (one asymptomatic subject was not tested). (PDF) [file pone.0244855.s006.pdf]

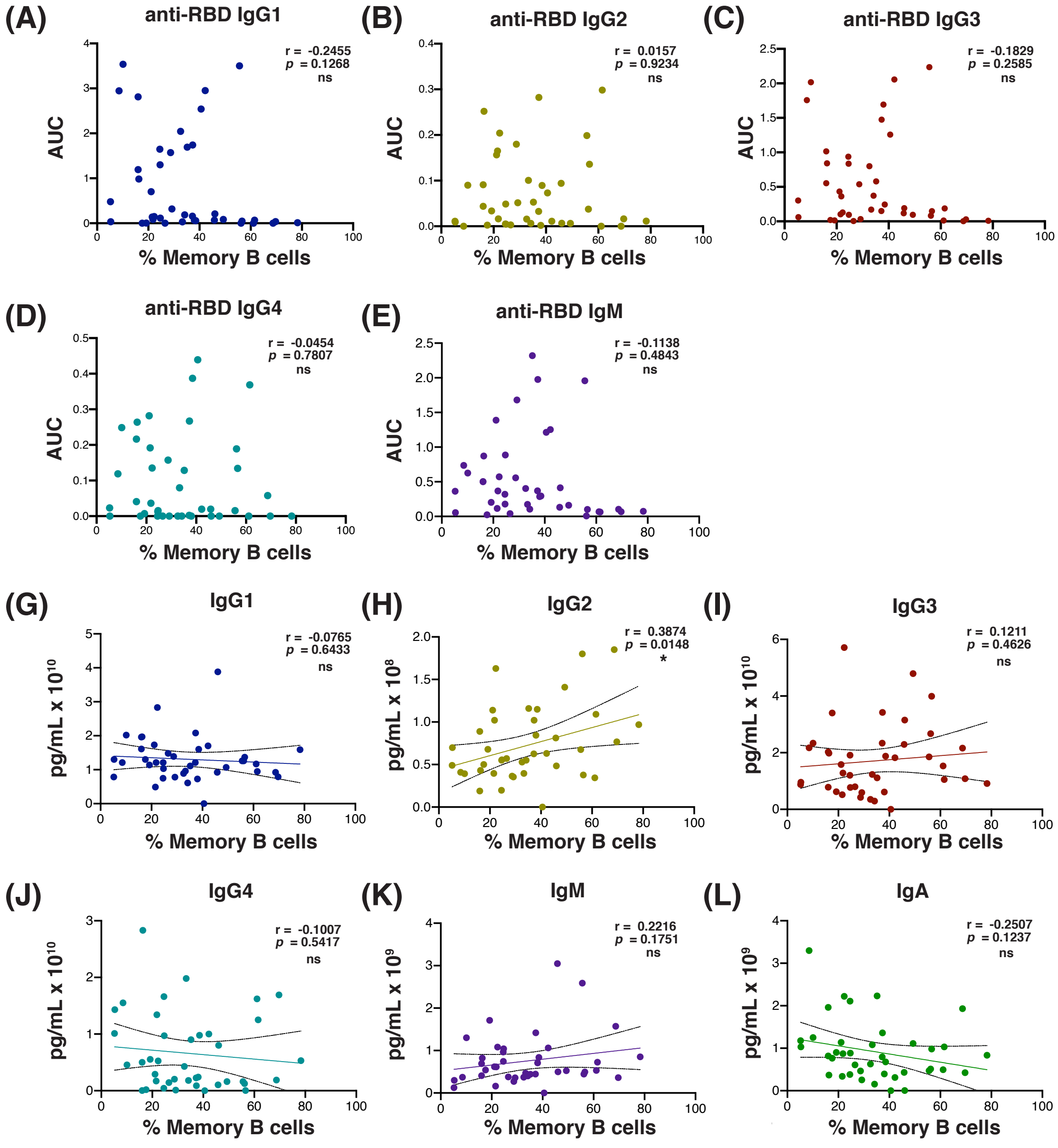

S6 Fig. Memory B cell frequency vs. anti-RBD antibody and total immunoglobulin in full cohort of convalescent plasma donors. (A-E) Scatterplot correlation of area under the curve for anti-RBD plasma antibody absorbance vs. memory B cell frequency for individual Ig isotypes and subclasses. (G-L) Scatterplot correlation of total plasma antibody concentration vs. memory B cell frequency from all convalescent subjects. Units reflect relative abundance of isotype, and were adjusted for graphical consistency. Pearson's correlation *r* value and 95% confidence intervals shown with two-tailed *p* value, alpha = 0.05. *n* = 40 for all anti-RBD plots, *n* = 39 for total Ig plots (one asymptomatic subject was not tested).
