# Supplementary material for: Switched and unswitched memory B cells detected during SARS-CoV-2 convalescence correlate with limited symptom duration
Source: PLoS One. 2021 Jan 28;16(1):e0244855. doi: 10.1371/journal.pone.0244855 (PMC7843013; doi:10.1371/journal.pone.0244855)
Supplement: S7 Fig — (A-B) Scatterplot correlation of area under the curve for plasma anti-RBD (A) IgG1 and (B) IgM absorbance vs. symptom duration. (C) Scatterplot correlation of symptom duration in days vs. frequency of (C) plasmablasts, (D) activated naive/memory, and (E) T-bet+ cells among CD19+ B cells. Scatterplot correlation of symptom duration vs. age in years. n = 11 for all plots, circled in Fig 3A. Spearman’s correlation rs value and 95% confidence intervals shown with two-tailed p value, alpha = 0.05 for all analyses. Regression lines shown to demonstrate trend only. (PDF) [file pone.0244855.s007.pdf]

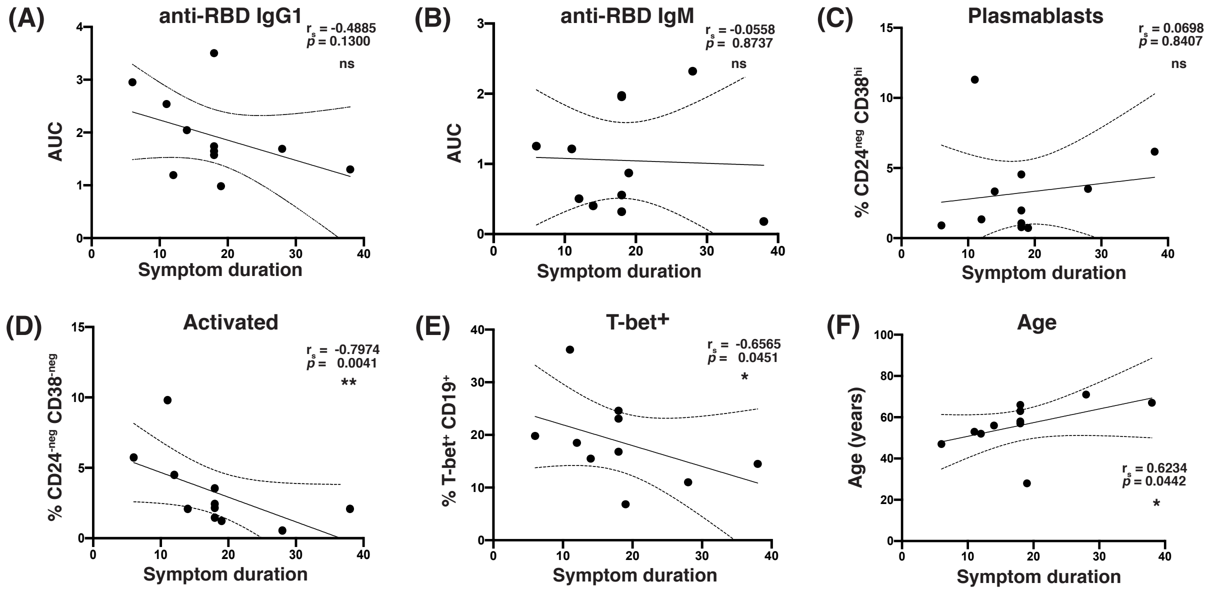

S7 Fig. Symptom duration correlations in anti-RBD IgG1-producing convalescent plasma donors. (A-B) Scatterplot correlation of area under the curve for plasma anti-RBD (A) IgG1 and (B) IgM absorbance vs. symptom duration. (C) Scatterplot correlation of symptom duration in days vs. frequency of (C) plasmablasts, (D) activated naive/memory, and (E) T-bet+ cells among CD19+ B cells. Scatterplot correlation of symptom duration vs. age in years. n = 11 for all plots, circled in Fig 3A. Spearman's correlation r_S value and 95% confidence intervals shown with two-tailed p value, alpha = 0.05 for all analyses. Regression lines shown to demonstrate trend.
